# Preparation and Characterization of Antibacterial Polypropylene Meshes with Covalently Incorporated β-Cyclodextrins and Captured Antimicrobial Agent for Hernia Repair

**DOI:** 10.3390/polym10010058

**Published:** 2018-01-11

**Authors:** Noor Sanbhal, Ying Mao, Gang Sun, Yan Li, Mazhar Peerzada, Lu Wang

**Affiliations:** 1 Key Laboratory of Textile Science and Technology of Ministry of Education, Room 4023, College of Textiles, Donghua University, 2999 North Renmin Road, Songjiang, Shanghai 201620, China; Noor.Sanbhal@faculty.muet.edu.pk (N.S.); Maoying-dhu@163.com (Y.M.); gysun@ucdavis.edu (G.S.); 2 Department of Textile Engineering, Mehran University of Engineering and Technology, Jamshoro 76062, Sindh, Pakistan; Mazhar.peerzada@faculty.muet.edu.pk; 3 Division of Textiles and Clothing, University of California, Davis, CA 95616, USA

**Keywords:** antibacterial meshes, guest molecule, cyclodextrin grafting, polypropylene, oxygen plasma

## Abstract

Polypropylene (PP) light weight meshes are commonly used as hernioplasty implants. Nevertheless, the growth of bacteria within textile knitted mesh intersections can occur after surgical mesh implantation, causing infections. Thus, bacterial reproduction has to be stopped in the very early stage of mesh implantation. Herein, novel antimicrobial PP meshes grafted with β-CD and complexes with triclosan were prepared for mesh infection prevention. Initially, PP mesh surfaces were functionalized with suitable cold oxygen plasma. Then, hexamethylene diisocyanate (HDI) was successfully grafted on the plasma-activated PP surfaces. Afterwards, β-CD was connected with the already HDI reacted PP meshes and triclosan, serving as a model antimicrobial agent, was loaded into the cyclodextrin (CD) cavity for desired antibacterial functions. The hydrophobic interior and hydrophilic exterior of β-CD are well suited to form complexes with hydrophobic host guest molecules. Thus, the prepared PP mesh samples, CD-TCL-2 and CD-TCL-6 demonstrated excellent antibacterial properties against *Staphylococcus aureus* and *Escherichia coli* that were sustained up to 11 and 13 days, respectively. The surfaces of chemically modified PP meshes showed dramatically reduced water contact angles. Moreover, X-ray diffractometer (XRD), differential scanning calorimeter (DSC), and Thermogravimetric (TGA) evidenced that there was no significant effect of grafted hexamethylene diisocyanate (HDI) and CD on the structural and thermal properties of the PP meshes.

## 1. Introduction

Synthetic meshes have been used with the objective of delivering long-term reinforcement to the damaged tissues of the abdominal hernia [1]. An abdominal hernia is a “protrusion or rupture” of an organ through the abdomen wall, causing defect in the abdomen [2]. Numerous studies demonstrated that, among synthetic meshes, non-absorbable polypropylene (PP) warp-knitted, lightweight, and large pore size meshes are the most widely used implants to reduce hernia recurrence [3,4,5,6,7], due to their chemically inert property, light weight, excellent tissue incorporation ability, hydrophobicity, and stability that can withstand a maximum abdominal pressure of 170 mm Hg. The main drawback of these implants is that all synthetic devices favor infection due to the uneven topography of knitted meshes [8]. Thus, these medical mesh devices are prone to bacteria growth and biofilm formation [9,10]. PP mesh infection is a serious surgical complication and is one of the main causes of hernia repair failure [11]. The majority of the bacteria concerned in surgical wound infections are *Staphylococcus aureus* (SA). Mesh infection is difficult to cure when the infected wound is colonized. Mesh infection not only causes removal of infected mesh but health care costs and risks of morbidity and mortality are also increased after mesh infection [12].

One way to minimize mesh infection is pre-operative antibiotic prophylaxis or pre-soaking of mesh in antibiotics during hernia surgery [13]. Nevertheless, PP is a non-absorbable material; even after decades of such antibiotic treatments, mesh infection has remained a major problem [14,15]. Another way to control surgical site infection (SSI) may be to deliver a heavy dose of antibiotics by injections or orally in clinic. However, the distribution of such heavy antibiotic doses to the whole body causes severe side effects. Therefore, sustained release of antibiotics for a suitable duration might be the key to avoiding post-operative mesh infection [16].

The use of cyclodextrin (CD) in the designed preparation is due to its extraordinary properties of hydrophilicity, biocompatibility, and efficient sustained captive and delivery nature. Cyclodextrin monomers like β-CD are renowned as the most accessible cyclic oligosaccharides with unique properties to form complexes with drug molecules without covalent links in their hydrophobic cavities. CDs can be commonly cross-linked with various reactants due to their chemical structure, which contains multiple hydroxyl groups. Thus, they have the advantage of reacting with hexamethylene diisocyanate (HDI) [17].

Triclosan is a proven antibacterial agent with properties similar to antibiotics, with low solubility in water [18]. However, there have been concerns about the use of triclosan in medical devices. Nevertheless, the application of triclosan as a model antimicrobial agent can still present the designed functions of the chemically modified polymers and triclosan has been used to be captured into the system [19,20].

In the past, polypropylene nonwoven implant devices have been finished with cyclodextrin at a higher temperature and loaded with antibacterial substances for mesh infection prevention [21], or surface activation of PP devices with atmospheric pressure plasma and subsequent coating of chitosan/ciprofloxacin has been performed for four days of antibacterial release [22]. The selection of material type (lightweight knitted meshes) is a key factor for hernia repair and in later cases chitosan could deliver antibacterial properties for a limited time.

Cold oxygen plasma is a suitable surface treatment process to modify the surfaces of polymers without changing their bulk properties [23]. Thus, low-pressure cold oxygen plasma treatment is one of the best approaches available so far to increase the adhesion and wettability of polymer surfaces [24], and also has good reproducibility [25]. Grafting reactions onto PP fibers’ surfaces could be introduced by surface activation with oxygen plasma to change the chemistry of PP fibers’ surfaces, which could also increase the wettability and adhesion for grafting [26]. Overall, the PP surface is prone to oxygen plasma treatments and results in C–H bond cleavage, which may lead to the introduction of carboxyl and hydroxyl groups [27,28]. These results suggest that low-pressure cold oxygen plasma is an effective pretreatment process to modify the surfaces of PP fibers. To the best of our knowledge, no study has been published to graft β-cyclodextrin onto PP knitted mesh devices for mesh infection prevention. However, chemical covalent connection of cyclodextrin onto the inert polypropylene mesh fibers is a key step in the modification reactions.

**Figure 1:**
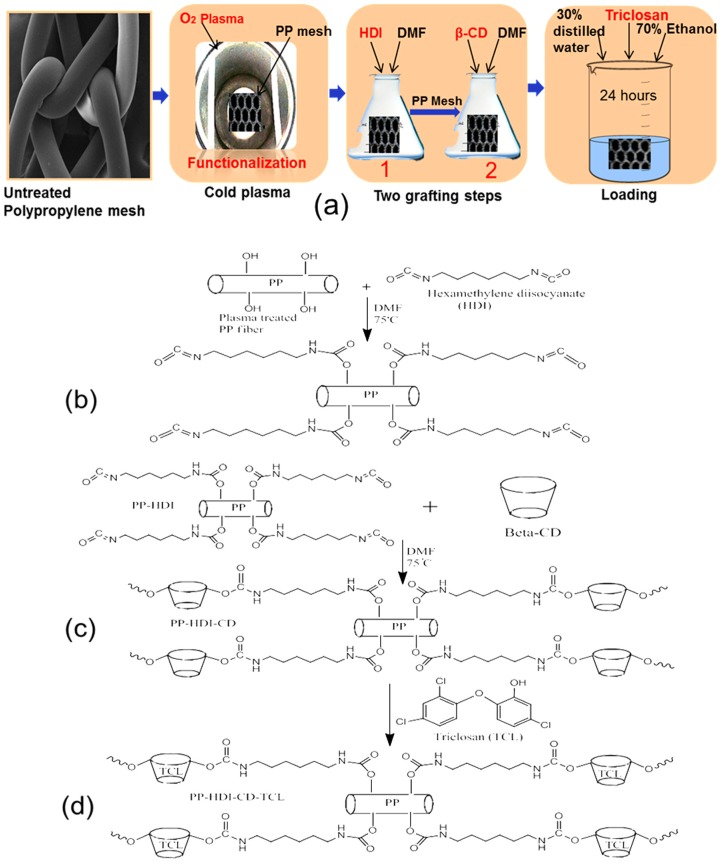
(**a**) Illustrations of experimental design for β-CD grafted antibacterial surgical PP meshes; Schemes (**b**) step-1, PP-HDI grafting; (**c**) step-2, PP-HDI-CD grafting; and (**d**) triclosan loaded meshes.

Therefore, the aim of this study was to prepare cyclodextrin-grafted PP meshes, which could make complexes with triclosan and demonstrate sustained antibacterial release for mesh infection prevention. For this reason, low-pressure cold oxygen plasma was used to functionalize the surfaces of PP fibers to create a hydroxyl or carboxyl group on PP surfaces before two simple grafting steps were performed. In the first step, hexamethylene diisocyanate (HDI) reacted with plasma-activated PP meshes and in the second step CD covalently bonded with PP-HDI meshes. Moreover, grafted CD samples were loaded with triclosan for antibacterial released function, as illustrated in Figure 1a. Characterization of modified PP meshes such as surface morphology, element analysis, and structural, thermal, and hydrophilicity were investigated using scanning electron microscopy (SEM), Energy Despersive X-ray spectroscopy (EDX), Fourier Transform Infrared Spectroscopy (FTIR), X-ray diffractometer (XRD), differential scanning calorimeter (DSC), and Thermogravimetric (TGA). Additionally, antibacterial properties were assessed using a suitable sustained efficacy test. Thus, results showed that CD was successfully grafted onto the surfaces of PP meshes and captured triclosan. Moreover, modified PP mesh devices provided prolonged and stable antimicrobial properties without affecting the original properties of PP mesh devices, which could be used in the prevention of mesh infection.

## 2. Materials and Methods

### 2.1. Materials

Polypropylene (PP) warp-knitted mesh, made of fine diameter (0.1 mm) monofilament with a large pore size (3.5 mm × 2.5 mm) and light weight (27 g/m^2^), was received from Nantong Newtec Textile and Chemical Fiber Co. Ltd., Nantong, China. For water contact angle measurement, a PP nonwoven melt-blown fabric of 23 g/m^2^ wasn selected. Hexamethylene diisocyanate (HDI) (≥98%) CAS: 822-06-0 and β-Cyclodextrin (CD) (≥97%) CAS: 7585-39-9 was purchased from Sigma Aldrich, Shanghai, China. Triclosan CAS: 3380-34-5 was purchased from Aladdin Company Shanghai, China, while *N*-*N* dimethylformamide (DMF) Case No. 68-12-12 was received from Shanghai Ling feng Company. Ethanol (≥99.7%) was purchased from Yangyuan Chemical Engineering, Changshu, Jiangsu, China.

### 2.2. Methods

#### 2.2.1. Cold Plasma Surface Functionalization

A cold plasma machine (HD-300) at low pressure was used for the surface functionalization of PP mesh samples. The machine is consisted of Power (500 W), radio frequency (13.56 MHz) of plasma and a vacuum reaction chamber (300 cm × 300 cm) for the treatment of samples. All PP mesh samples (10 cm × 10 cm) were surface activated at constant powered (40 W) and pressure (10 pa) of oxygen gas. Moreover, optimized treatment time (300 s) was selected for all samples.

#### 2.2.2. Two Grafting Steps and PP-HDI-CD Incorporation

Plasma treated PP meshes was grafted with β-cyclodextrins by simple two grafting step method. First HDI (2–6%) was reacted with the plasma treated samples. Afterwards, the surface modified PP-HDI samples were further reacted with CD (2–8%). For both processes the liquor ratio was (mesh:solution) 1:100. The time and temperature were 30–90 min and 65–85 °C, respectively. Afterward, the PP surface modified meshes (HDI-CD) were washed several times with warm distilled (50 °C) water to eliminate residues of unconnected chemical before drying. HDI grafted PP samples were named PP-HDI-2 (2%), PP-HDI-4 (4%), PP-HDI-6 (6%) and CD-HDI modified polypropylene mesh samples were respectively named as HDI-CD-2 (2%), HDI-CD-4 (4%), HDI-CD-6 (6%), and HDI-CD-8 (8%) according to the CDs concentrations in the solutions.

#### 2.2.3. Loading of Triclosan

Cyclodextrin (HDI-CD)-modified samples (0.5 g) were immersed in the 50 mL mixture (70% ethanol and 30% water) containing 0.6% (0.3 g) triclosan as a guest molecule. All samples were soaked for 24 h before drying. The modified polypropylene samples after triclosan loading were named as CD-TCL-2, CD-TCL-4, and CD-TCL-6, and CD-TCL-8, respectively.

### 2.3. Characterization Techniques

#### 2.3.1. SEM and EDX

A coating machine (E-1045, Hitachi, Tokyo, Japan) was first used to coat untreated and modified samples with platinum (pt.) ion. Subsequently, all coated samples were tested by a scanning electron microscope (JEOL JSM 6330, Tokyo, Japan) and Energy Despersive X-ray spectroscopy (EDX) (Oxford Instrument ISIS 300, Oxfordshire, UK) connected with SEM.

#### 2.3.2. FTIR

Chemical compositions of untreated and modified samples were measured by a Fourier Transform Infrared Spectroscopy (FTIR) Attenuated Total Reflection mode (ATR) (Nicolet 6700, Downers Grove, IL, USA). All samples were characterized with resolution of 4.0 cm^−1^ and wave number ranging from 500 to 4000 cm^−1^.

#### 2.3.3. Contact Angle

Due to the fine filament diameter (0.1 mm) and larger pore size of the PP meshes, a nonwoven PP was preferred as a model fabric to reflect the differences between the contact angles of the PP control and the modified meshes. Two methods, captive bubble (static contact angle) and sessile drop (dynamic contact angle), were used to measure the water contact angle, using WCA 20 software to estimate the hydrophilic/hydrophobic behavior of samples. A Teli CCD camera (Data physics instrument, San Jose, CA, USA) was used to capture images of water contact angle and at least five drops of 5 µL for each sample were added to get an average contact angle.

#### 2.3.4. XRD

Powders of PP control and modified samples were prepared for X-ray diffractometer (XRD) examination. An XRD machine (Rigaku D/MAX 2550/PC, Tokyo, Japan) was used for scanning rate of 0.02/min at 40 kV, 200 mA and a range of 0°–60° (2ϴ) to characterize all samples.

#### 2.3.5. Thermal Analysis

PP control and modified mesh samples were scanned on a differential scanning calorimeter (DSC) (Perkin Elmer 4000, Downers Grove, IL, USA) at a rate of 20 °C /min over the range of 20–250 °C; the cooling rate was set to 20 °C/min.

Thermogravimetric analysis was conducted to evaluate the percentage residual weight loss of PP control and modified samples. A Perkin Elmer thermogravimetric analyzer (TGA) 4000 (Downers Grove, IL, USA) device was used to scan all samples over a temperature range of 250–550 °C at 30 °C/min. Thus, nitrogen gas was used at a flow rate of 20 mL/min with a pressure of 2 bars during characterization.

#### 2.3.6. Antibacterial Activity Assessment

##### Agar Diffusion Test Method (Qualitative Analysis)

Antibacterial activity was conducted using the agar diffusion test method described in a previously published research paper [16]. First, 400 µL of bacterial suspension (1 × 10^8^ CFU/mL) (*Staphylococcus aureus* ATCC 25923 and *Escherichia coli* ATCC25922) was evenly spread on to the agar plate with the help of pre-sterilized disposable swabs. Then, PP control and modified samples of size 1 cm × 1 cm were placed on an agar plate and incubated for 24 h at 37 °C. The zone of inhibition of incubated samples was measured with a digital vernier caliper in four directions and reported as the mean value. The zone of inhibition diameter was measured by the following formula:

*L* = (*D* − *T*)/2

where *L* = zone of inhibition, *T* = diameter of original sample, and *D* = inhibition zone diameter after 24 h incubation.

##### Sustained Efficacy Test (Serial Plate Transfer Test)

The objective of this test was to evaluate the antibacterial activity of CD-grafted samples with reference to the antibacterial release time in days. After each 24 h, the agar plate was changed and mesh samples (1 cm × 1 cm) were transferred to a new agar plate containing similar colony forming units (1 × 10^8^ CFU/mL) of bacteria (*Staphylococcus aureus ATCC 25923* and *Escherichia coli ATCC25922*) after contact with the previous one. The test was continued until modified meshes sustained their antibacterial activity.

#### 2.3.7. Statistical Analysis

The data in the figures are described as mean and standard deviations. However, the error bars in the figures indicate standard deviations. ANOVA one-way analysis (single-factor) was used to determine the differences between each sample and the data in the figures are labeled, such as *p* < 0.001 (***), *p* < 0.01 (**), and *p* < 0.05 (*). Although a *p* value of (*) < 0.05 was considered as the confidence interval, variances were first confirmed to be significant.

## 3. Results and Discussion

### 3.1. Cyclodextrin Grafting and Loading of Triclosan

The grafting of β-cyclodextrin (CD) onto the PP fibers was achieved by a two-step reaction, as shown in Figure 1. Before grafting, low-pressure oxygen plasma was used to create a hydroxyl group on the surfaces of the PP meshes. Afterward, two grafting steps were performed. In first step hexamethylene diisocyanate (HDI) was reacted (Figure 1b) with the plasma-treated PP fibers and then in second step PP-HDI samples were further reacted with hydroxyl groups of β-CD (Figure 1c). However, cyclodextrins are well suited to form complexes with triclosan. Thus, triclosan was loaded (Figure 1d) into HDI-CD grafted samples.

**Figure 2:**
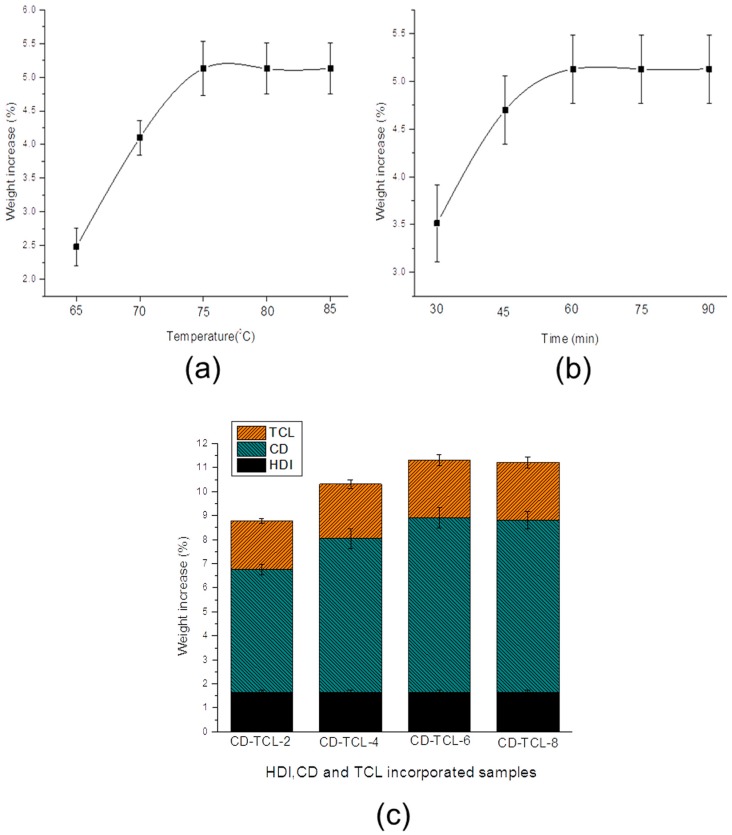
Optimization of two-step grafted PP meshes; (**a**) Temperature for grafting; (**b**) reaction time; (**c**) weight increase % of HDI-CD-TCL samples. Data of each sample expressed as averages (*n* = 3).

The process parameters, reaction time, and temperature of the cyclodextrin grafting reaction (Figure 2) were optimized. The effect of temperature on CD grafting (Figure 2a) indicates that weight % increased with an increase in temperature from 65 to 75 °C. However, an additional temperature increase from 75 to 85 °C did not have any effect on the weights of the treated samples. Grafting reaction time was also optimized, as illustrated in Figure 2b, and the grafted weight of the products increased with an increase in reaction time from 30 to 60 min. Thus, a temperature of 75 °C and time of 60 min were considered the optimum conditions for HDI and cyclodextrin grafting. Moreover, Figure 2c displays the total weight increase for HDI, CD, and triclosan. HDI (6%) showed better results for the grafting reaction on to the PP meshes and demonstrated a total weight increase of 1.63%. All CD-grafted samples were treated with the same concentration of HDI. However, it was difficult to find the real increased weight of the HDI samples due to the fact that PP-HDI samples were advanced to the second step of CD grafting without washing to save the unreacted diisocyanate group on the HDI-treated PP surface. The average weight increases of CD grafting for HDI-CD-2, HDI-CD-4, HDI-CD-6, and HDI-CD-8 were 5.130%, 6.410%, 7.27%, and 7.17%, respectively. Nevertheless, after triclosan loading the average corresponding weight increased to 2.025%, 2.268%, 2.414%, and 2.40%, respectively. HDI-CD-6 was considered the best sample, which gained more weight of CD and triclosan. Thus, CD-HDI-6 and CD-TCL-6 samples were included in further testing. Moreover, PP meshes without plasma treatment were also used for HDI grafting, but unfortunately we did not succeed with HDI grafting. Therefore, plasma treatment was key to increasing the surface roughness of PP meshes for better grafting of HDI.

### 3.2. Surface Morphology of PP Meshes

**Figure 3:**
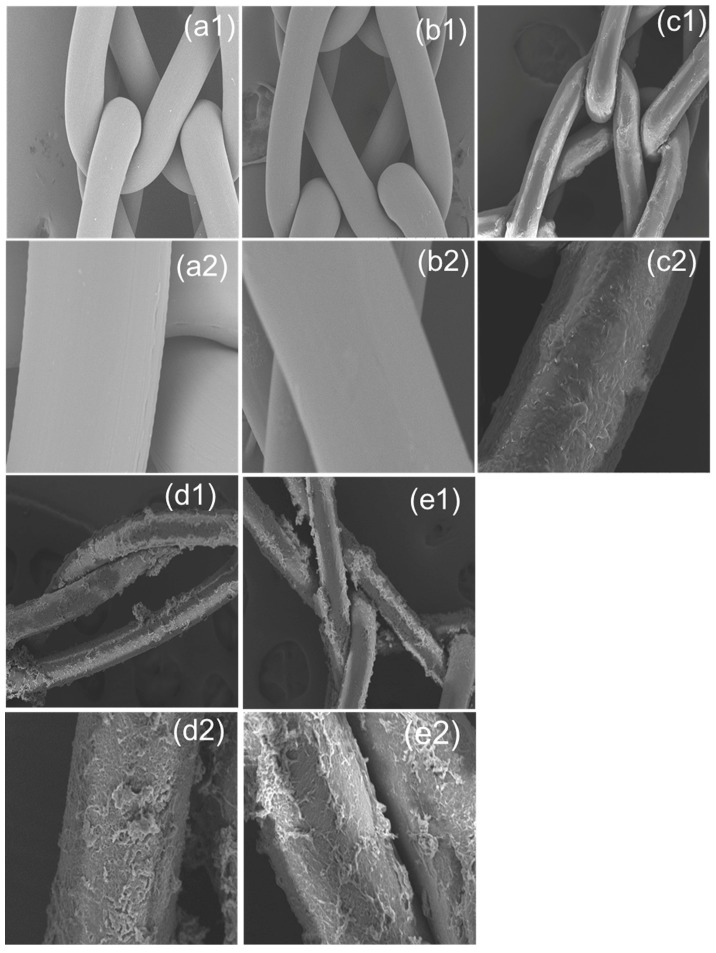
SEM micrographs; (**a1**,**a2**) PP control; (**b1**,**b2**) oxygen plasma treated; (**c1**,**c2**) HDI grafted PP-HDI-6; (**d1**,**d2**) HDI-CD-6; and (**e1**,**e2**) CD-TCL-6 (top row 200×, bottom row 1000×).

Polypropylene (PP) knitted mesh surfaces before plasma treatment (Figure 3a1,a2) were very smooth, but after plasma treatment (Figure 3b1,b2) the PP surface became slightly rough. The samples after the plasma treatment and reaction with HDI showed a thin layer of coating (Figure 3c1,c2) on the surfaces of PP fibers. Moreover, the surfaces of the HDI-CD grafted samples (Figure 3d1,d2) are covered with small hills and valleys. It can be observed (Figure 3e1,e2) that after triclosan loading the HDI-CD grafted samples showed similar but a little more swollen coating on the PP fibers, indicating that the loading of triclosan did not affect the grafted structure on the PP fibers.

Fauland reported that low-pressure oxygen plasma can be a suitable pretreatment process to introduce polar groups onto the surfaces of PP fibers and increase their wettability and adhesion for improved grafting of hexamethyl-disiloxane (HMDSO) [26]. Similarly, using low-pressure cold oxygen plasma, HDI-CD structures are successfully grafted onto the chemically inert PP surfaces. The SEM results confirmed that a layer of HDI coated was further connected with β-CD.

### 3.3. Characterization of HDI, CD, and Triclosan on Modified PP Mesh

**Figure 4:**
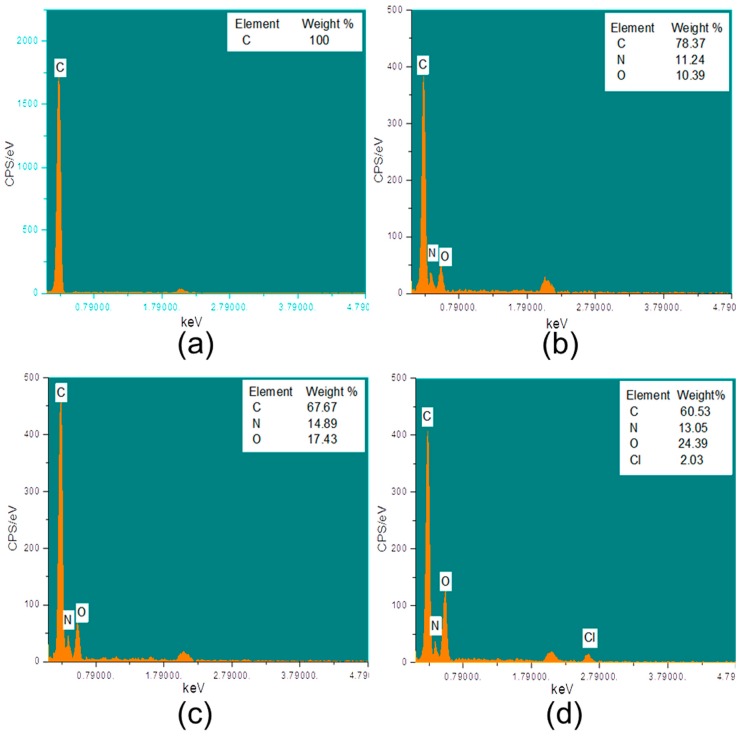
EDX spectra and atomic percent of PP control and modified meshes (**a**) PP control; (**b**) HDI grafted, PP-HDI-6; (**c**) HDI-CD-6; (**d**) CD-TCL-6.

The PP control and grafted samples were characterized using EDX (Figure 4) for elemental analysis.

It can be observed that the PP control (Figure 4a) sample shows the presence of a (C) carbon atom that is within 0.5 keV. Nevertheless, PP-HDI-6 (Figure 4b) shows two additional peaks (O) and (N) in addition to the carbon atom and all three peaks are under 0.5 keV, confirming the presence of hexamethylene diisocyanate (HDI) on PP surfaces. Moreover, HDI-CD-6 displayed the existence of the same carbon (C), oxygen (O), and nitrogen (N) (Figure 4c) but at different peak heights, which confirmed the presence of cyclodextrin. It can be observed that CD-TCL-6 showed (Figure 4d) an additional peak of chlorine (Cl) atom within 3.0 keV in addition to carbon, nitrogen, and oxygen atoms, confirming the successful loading of triclosan onto the CD-grafted samples.

**Figure 5:**
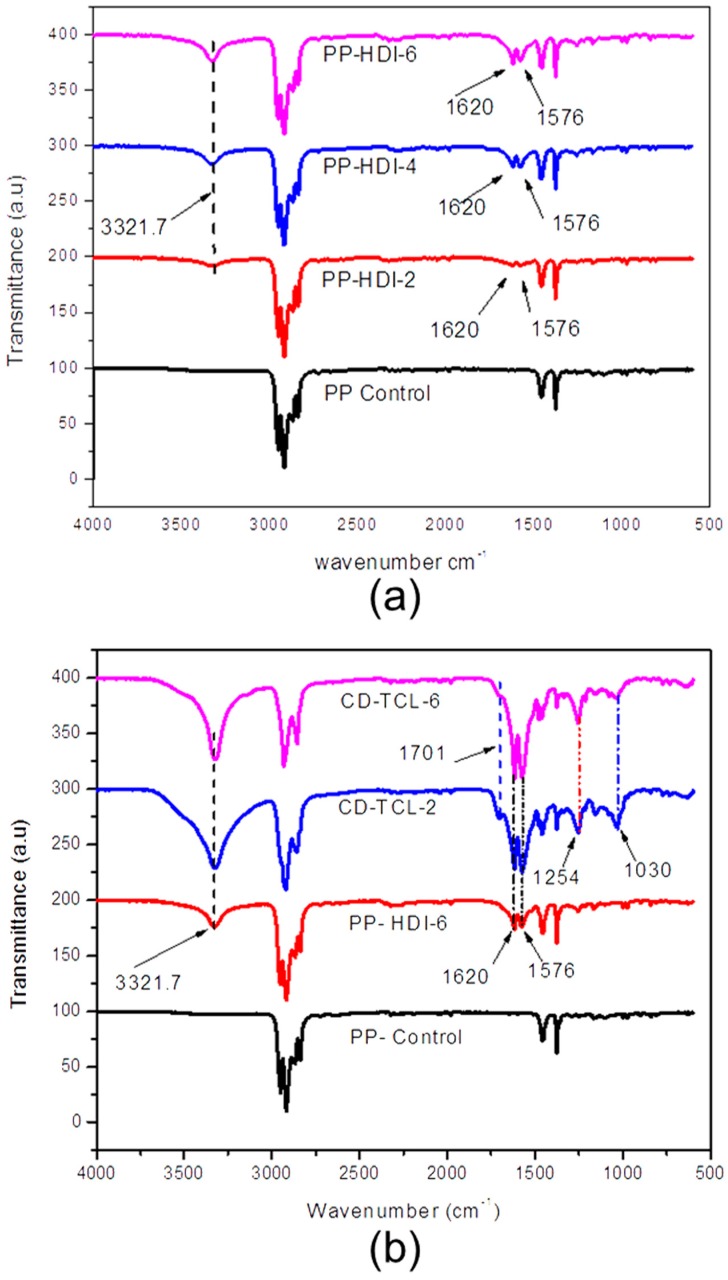
FTIR (ATR) spectra; (**a**) PP-HDI grafted samples (PP-HDI-2), (PP-HDI-4) and PP-HDI-6; and (**b**) PP control, PP-HDI-6-grafted, and triclosan-loaded (CD-TCL-2 and CD-TCL-6) samples.

Structures of the PP control, PP-HDI, and HDI-CD-grafted samples at different reaction stages were analyzed using FTIR-ATR. The PP control exhibits absorbance bands at 2950 cm^−1^ (va CH_3_), 2916 cm^−1^ (va CH_2_), 1452 cm^−1^ (δCH_3_), and 1376 cm^−1^ (δCH_3_) [29,30]. Nevertheless, all PP-HDI grafted samples (Figure 5a) showed additional absorbance peaks at 3321 cm^−1^ (OH), 1620 cm^−1^ (ν C=O), and 1576 cm^−1^ (δNH) due to hydroxyl, amide I, and amide II formations, respectively. CD-grafted (Figure 5b) polypropylene samples maintained all previous peaks of HDI-grafted samples and additionally showed absorbance peaks at 1701 cm^−1^ (carbonyl group, ν C=O) and C–H (vCH) stretch at 1256 cm^−1^. However, C–O groups of CD also appear at 1030 cm^−1^, consistent with a previously published paper [31]. The absorbance band of amide I 1620 cm^−1^ (ν C=O) and amide II 1576 cm^−1^ (δNH) can be also observed in all CD-grafted samples, which confirmed the formation of urethane, consistent with the literature on CD [17,32]. Moreover, we did not find any absorbance peak differences between HDI-CD (CD-grafted) and CD-TCL (triclosan-loaded) samples, which is due to the fact that triclosan was not reacted but captured by the CD cavity.

### 3.4. Hydrophilicity of PP Meshes (Water Contact Angle)

**Figure 6:**
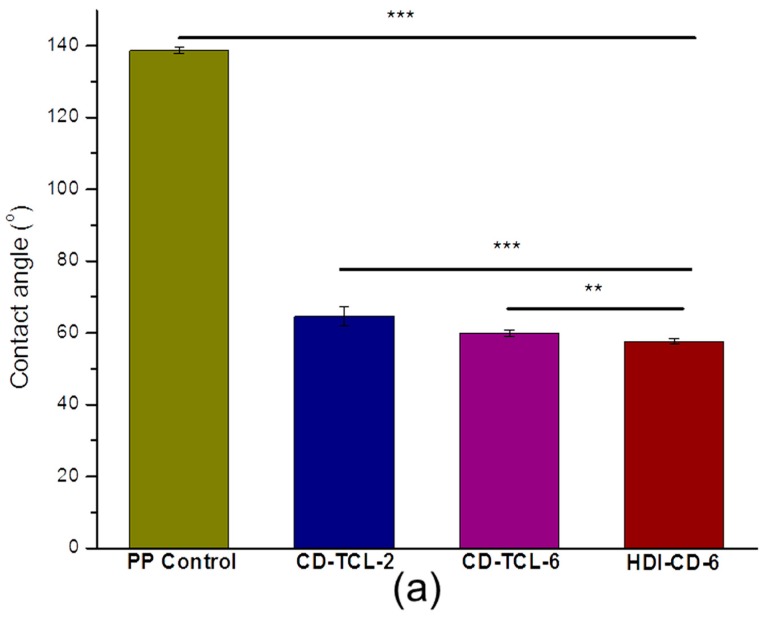

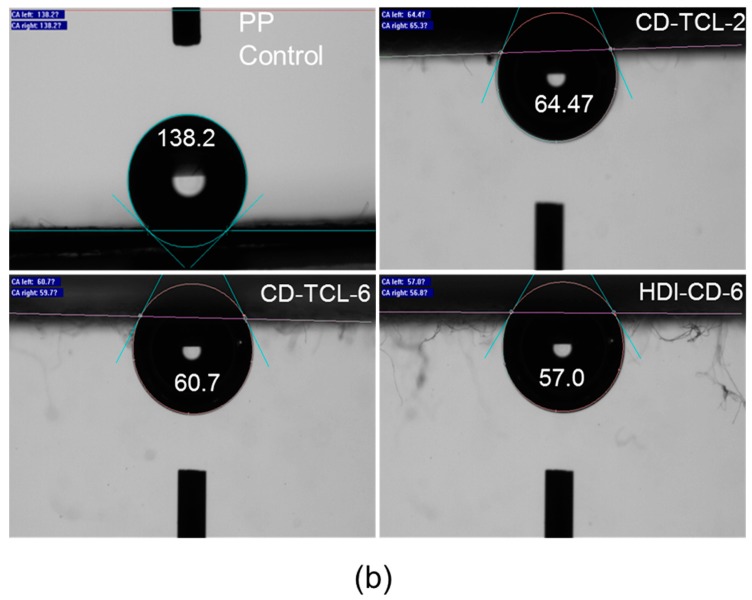
(**a**) Contact angles measured by sessile drop method and captive bubble (**b**) average contact angle drops, PP control (sessile drop), triclosan-loaded CD-TCL-2, triclosan-loaded CD-TCL-6 and CD-grafted HDI-CD-6. Data for each sample are expressed as an average of five measurements (*n* = 5). Statistical differences are indicated with (***) for *p* < 0.001 and (**) for *p* < 0.01.

Water contact angles of PP control and surface-modified samples (Figure 6) were measured by both captive bubble and sessile drop methods. After CD grafting, all modified samples became much more hydrophilic. Thus, the captive bubble method was mainly used to measure the water contact angles of the modified surfaces. Nevertheless, the PP control still retains a hydrophobic surface; therefore, sessile drop was used to measure its water contact angle. As shown in Figure 6a, the contact angles of all treated samples reduced dramatically. The average water contact angle of the PP control was 138.66° and decreased to 64.5° (113.3%), 59.82 (131.8%), and 57.7° (140.7%) for CD-TCL-2, CD-TCL-6, and HDI-CD-6, respectively.

The average water contact angle difference between CD-TCL-2 (64.57°) and CD-TCL-6 (59.82°) was 7.94% (*p* < 0.001), which reveals the effect of more CD grafted onto CD-TCL-6. Likewise, the difference between water contact angles of HDI-CD-6 (59.82°) and triclosan-loaded CD-TCL-6 (57.6°) was 3.84%. This indicates the hydrophobicity of host molecule triclosan loaded in comparison to plain CD. These results are in consensus with a paper published in 2017 [33]. The average water contact angle drops for the PP control and the grafted samples are shown in Figure 6b; it can be observed that CD-TCL-2 shows a slightly increased contact angle compared to CD-TCL-6 and HDI-CD-6.

β-cyclodextrin makes complexes with a wide range of molecules due to its cavity and multi-hydroxyl groups [34]. As expected, all cyclodextrin-grafted PP samples showed remarkably decreased water contact angles. Nevertheless, the hydrophobicity of grafted CD increases with the increase in complexes between CD and triclosan.

### 3.5. Structural and Thermal Properties

**Figure 7:**
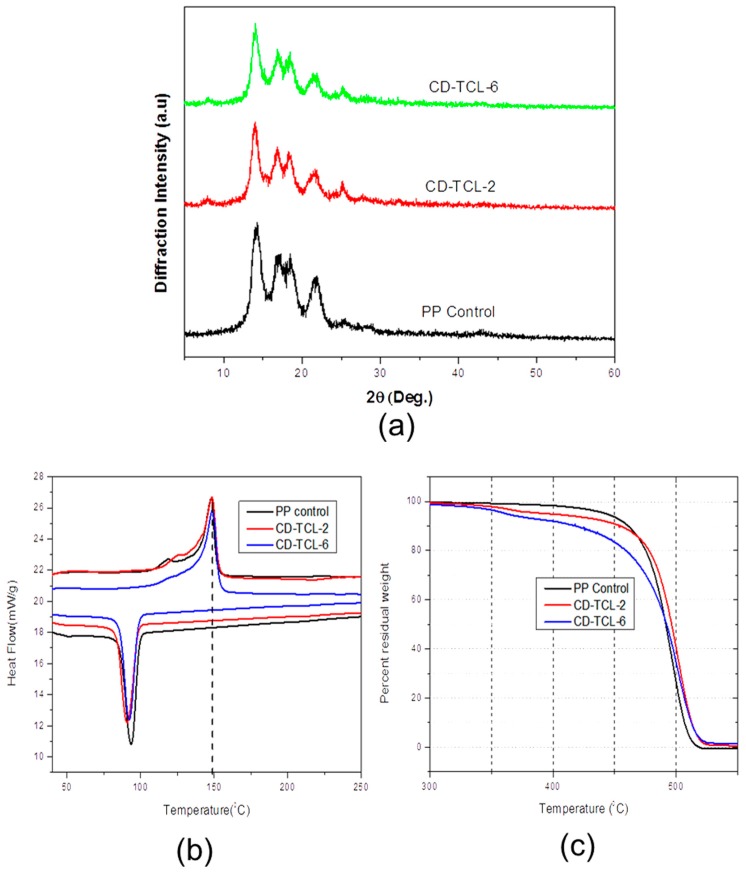
Structural and thermal properties: (**a**) XRD pattern of treated and untreated PP meshes; (**b**) DSC of PP control and modified samples; (**c**) TGA: percentage residual weight changes of PP control and modified samples.

The PP control and surface-modified samples were analyzed by XRD, as shown in Figure 7a. It can be observed that the PP control shows five peak lattices (14.21°, 17.14°, 18.93°, 21.40°, and 25°) up to 25° [35], and all modified samples maintained the same peaks and crystal structure. Furthermore, the crystallinity of CD-TCL-2 (61.30%) and CD-TCL-4 (63.04%) samples were slightly increased as compared to that of the PP control (61.05%). However, the change was minimal; therefore, cyclodextrin grafting has no significant influence on the crystal structure of the modified PP mesh devices.

As shown in Figure 7b, the PP control has a melting temperature of 148.08 °C, whereas CD-TCL-2 (148.52 °C) and CD-TCL-6 (148.90 °C) showed slightly increased melting temperatures due to HDI-CD grafting, consistent with a previously published research paper [21]. Similarly, the endothermic temperature for the PP control (94.2 °C) was slightly higher than CD-TCL-2 (90.9 °C) and CD-TCL-6 (92 °C). Figure 7c reveals the residual weight loss and thermal stability of the PP control and the grafted samples. It can be observed that the PP control was more thermally stable at a temperature of 400 °C and lost only 2% of its total weight. CD-TCL-2 and CD-TCL-6 were less thermally stable and lost their weights of 5% and 8% at the same temperature, consistent with a previously published paper [21]. This may be due to the less stable structure of CD on the grafted samples. Overall, there is no significant difference in the decomposition temperature of PP control and grafted samples.

### 3.6. Antibacterial Activity

**Figure 8:**
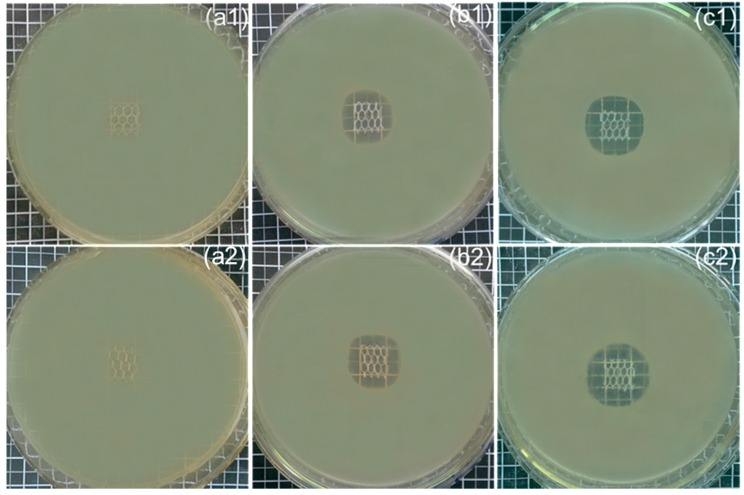
Inhibition zone diameter of (**a1**,**a2**) PP control, (**b1**,**b2**) CD-TCL-2, (**c1**,**c2**) CD-TCL-6, (top row) *S. aureus* and (bottom row) *E. coli*.

The antibacterial properties of the modified and PP control meshes were measured using qualitative analysis by inhibition zone diameter. Figure 8 displays the inhibition zone diameters of the untreated PP control and the modified (CD-TCL-2 and CD-TCL-6) meshes. The PP control and HDI-CD (without triclosan) samples did not show antibacterial properties. However, the PP-modified and triclosan-loaded samples demonstrated good inhibition zone diameters. However, CD-TCL-2 displayed an average inhibition zone of 5.06 and 5.133 mm for *S. aureus* and *E. coli,* respectively. CD-TCL-6 exhibited maximum inhibition zones of 6.2 and 6.40 mm to *S. aureus* and *E. coli*, respectively.

**Figure 9:**
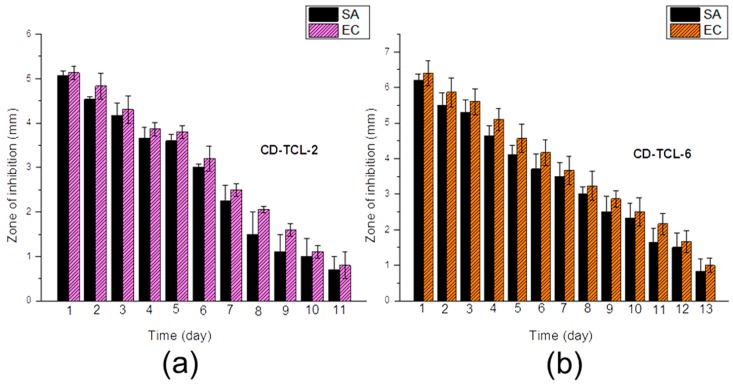
Sustained efficacy test (**a**) CD-TCL-2, (**b**) CD-TCL-6. Data for antimicrobial test samples are expressed as an average of three samples (*n* = 3).

Moreover, CD-TCL-2 and CD-TCL-6 (Figure 9) were further evaluated using a sustained efficacy tests to assess their durable antibacterial properties with respect to number of days. A sustained efficacy test is a very important test to analyze the release of antibacterial agent after each 24 h.

The average inhibition zone diameters of CD-TCL-2 and CD-TCL-6 for both bacteria (*E. coli* and *S. aureus*) decreased each day. CD-TCL-2 ended its antibacterial function after 11 days for both *S. aureus* and *E. coli*, and CD-TCL-6 ended its antibacterial function after 13 days. As shown in Figure 9a, CD-TCL-2 demonstrated different inhibition zones of diameters for both *S. aureus* and *E. coli.* It can be observed that *E. coli* samples demonstrated greater zones of inhibition diameters than *S. aureus* during all 11 days.

However, the zones of inhibition were decreased quickly after six days for both types of bacteria. The minimum inhibition zone for *S. aureus* and *E. coli* was 0.7 and 0.8 mm, respectively. In the case of CD-TCL-6 (Figure 9b), inhibition zone diameters of *S. aureus and E. coli* were also different to each other, and *E. coli* had greater inhibition zone diameters throughout all 13 days and the zone of inhibition decreased regularly with respect to days. The minimum inhibition zone for *S. aureus and E. coli* was 0.8 and 1 mm, respectively.

Overall, due to the fact that CD-TCL-6 samples carried more CD and triclosan as compared to the CD-TCL-2, CD-TCL-6 always demonstrated more powerful antimicrobial properties than CD-TCL-2. Generally, *E. coli* had a greater inhibition zone diameter for CD-TCL-2 and CD-TCL-6.

Previously, polypropylene implants have been coated with silver nano-clusters to prevent mesh infection [36]. However, the sustained release of antibacterial agent is a suitable method to ensure the release of antibiotic for a prolonged duration. Furthermore, Majumdar et al. also performed antibacterial properties for a polyester implant by (AATCC 100-2004) colony counting units a quantitative test method [37], which may not be sufficient to prevent mesh infection. Unfortunately, mesh infection is a major problem in hernia surgery and may require continuous antibacterial activity for at least 10 days. Thus, β-CD inclusion complexes with antibiotics could be a suitable technique to capture the antimicrobial agent and release it for a prolonged duration to prevent mesh infection. This work could be further carried out to perform in vitro and in vivo experiments.

## 4. Conclusions

This study proven that β-CD was covalently bonded onto PP mesh devices without affecting the original properties of the PP fibers. The products captured triclosan and provided excellent sustained released antibacterial properties. The low-pressure, cold oxygen plasma was able to activate the surfaces of PP meshes effectively, and created hydroxyl and carboxyl groups on PP surfaces for hexamethylene diisocyanate (DHI) grafting. HDI was successfully grafted on the oxygen plasma activated PP fibers’ surfaces. Moreover, β-CD was connected to the HDI-grafted PP meshes, and the modified PP meshes were successfully loaded with triclosan as a model antimicrobial agent. All samples were characterized using SEM, FTIR, EDX, XRD, TGA, DSC, and water contact angle.

## Supplementary Materials

The following are available online at www.mdpi.com/2073-4360/10/1/58/s1. Data for graphs and figures for the following are attached in a supplementary file in the form of CSV data. Graphs for Figure 2a–c, Figure 6a and Figure 9b.
